# Hydromyelia and Intramedullary Abscess Complications of Dermal Sinus Tract: Rare Case in a Child

**DOI:** 10.7759/cureus.33410

**Published:** 2023-01-05

**Authors:** Aldo J F da Silva, Bianca R dos Santos Silva

**Affiliations:** 1 Pediatric Neurosurgery Division, Santa Mônica Teaching Maternity - Alagoas State University of Health Sciences and General State Hospital, Maceio, BRA; 2 Nursing, University Hospital Professor Alberto Antunes, Maceio, BRA

**Keywords:** fever, mri, abscess, spina bifida occulta, child

## Abstract

The dermal sinus tract (DST) is an infrequent form of spina bifida occulta that can be complicated by infections. This is a case report of a child with a rare association of DST complications, namely hydromyelia and intramedullary abscess. A 1-year-old male child was admitted to the hospital with a history of fever and progressive loss of lower-limb movements and the presence of an ostium in the midline of the lumbar region and cutaneous stigmas with no secretion discharge. Computed tomography and magnetic resonance imaging (MRI) of the thoracolumbar spine revealed an extensive intradural T12-S1 abscess and cystic dilatation of the central medullary canal (hydromyelia) from T2 to T12. The surgery was conducted with the drainage of the large abscess and also improving hydromyelia. After 30 days of rehabilitation with physical therapy, the child could walk despite right crural monoparesis (possible L4/L5 injury). This report described a case of DST that showed a rare association of two complications, i.e., intramedullary abscess and hydromyelia, due to a late diagnosis. Sequelae could have been prevented with early diagnosis.

## Introduction

Dermal sinus tract (DST) is a rare form of spina bifida occulta that affects approximately one in every 2,500 live births [1]. It results from an incomplete separation of the neuroectoderm from the superficial ectoderm during embryogenesis, which occurs between the third and eighth weeks of gestation [2]. This causes the formation of a tract lined with multilayered epithelium, extending inward from the skin or subcutaneous tissues and possibly ending in the spinal elements (6%-7%), extradural space (10%-20%), or intradural space (58%-60%) [3]. DST may be present in the midline from the nasium to the coccyx, but it is most often located in the lumbar region [2].

This is a case report of a child who had a rare combination of DST complications, namely hydromyelia and intramedullary abscess.

## Case presentation

**Figure 1:**
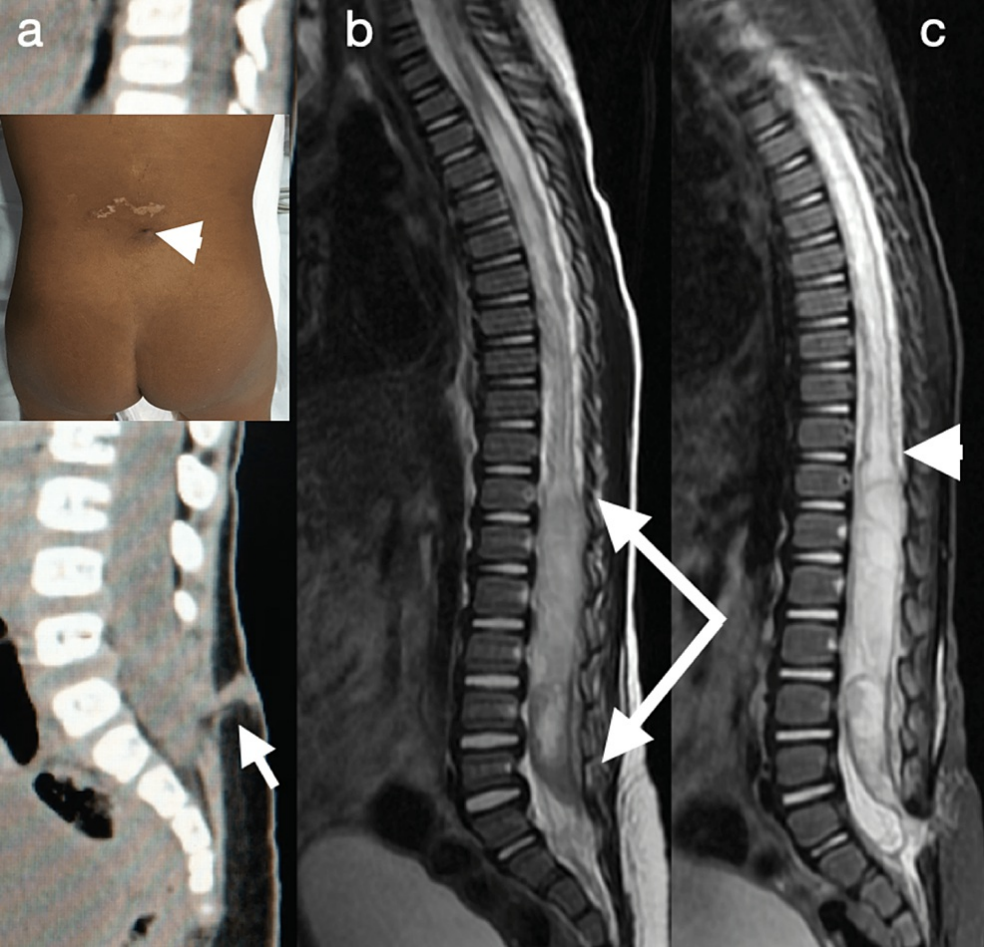
Ostium, computed tomography, and magnetic resonance imaging (a) Ostium in the midline of the lumbar region (white arrowhead) and computed tomography of the lumbosacral spine and sagittal section revealing a thickening of the vertebral canal, failure in the fusion of the posterior arches and a dermal sinus tract (white arrow); (b) and (c) magnetic resonance imaging, sagittal T2 and STIR (Short Time Inversion Recovery) showing evidence of hypersignal with an intradural lesion from T12 to S1 (white arrows) and cystic dilation of the central medullary canal from T2 to T12 (white arrowhead)

**Figure 2:**
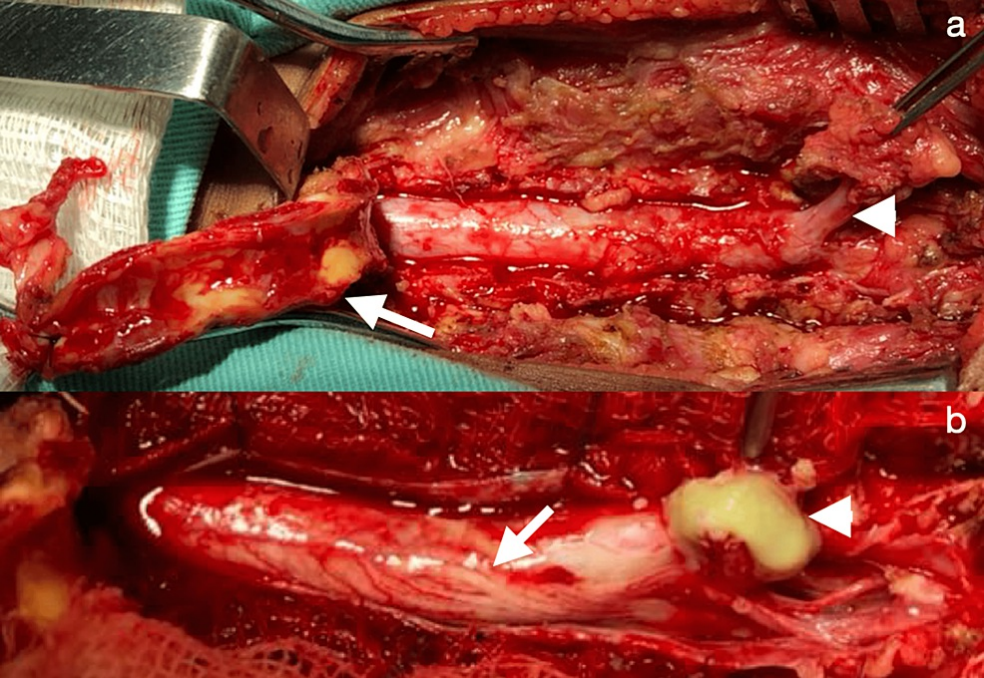
Intraoperative (a) Osteoplastic laminotomy (white arrow) with visualization of the dermal sinus tract (white arrowhead); (b) opening of the dural sac with visualization of the roots (white arrow) and drainage of intramedullary purulent discharge (white arrowhead).

A 1-year-old male infant was admitted to the emergency hospital with a fever (39°C) and difficulty moving his legs. His mother reported that the fever had started a week prior to hospitalization. Progressive loss of leg movements had begun three days before admission. On physical examination, the patient was crying and paraplegic (reduced tone and zero degree of muscle power) and an ostium was observed in the midline of the lumbar region (Figure 1a), along with cutaneous stigmas but no secretion discharge. Lumbar puncture for analysis of cerebrospinal fluid (CSF) was not possible. Computed tomography (CT) of the thoracolumbar spine revealed a thickening of the spinal canal with evidence of spina bifida and a dermal tract (Figure 1a). Magnetic resonance imaging (MRI) revealed that the sagittal T2 and short tau inversion recovery sequences showed an extensive intradural lesion with T12-S1 hypersignal, signs of a trapped spinal cord with a low conus medullaris at S1, cystic dilatation of the central medullary canal (hydromyelia) from T2 to T12 (Figures 1b-1c), a posterior fusion defect of the sacral spine and the presence of a dermal tract. The patient underwent a T12-L5 osteoplastic laminotomy (Figure 2a) followed by a durotomy (Figure 2b), which resulted in the drainage of a bulky abscess and improvement of the hydromyelia. *Staphylococcus aureus* and *Escherichia coli* were grown on culture. For treatment, intravenous therapy with ceftriaxone and vancomycin was chosen for a period of four weeks. On the second postoperative day, the child was able to move his lower limbs. After 30 days of rehabilitation with physical therapy, the child could walk despite right crural monoparesis (possible L4/L5 injury).

## Discussion

Hart described the first case of an intramedullary abscess in 1830 [4]. Infection caused by DST may be diffuse as meningitis or focal as epidural, subdural, or parenchymal abscess [5]. In this paper, we present the case of a patient with DST in the lumbar region, originating in the midline and complicated by an intramedullary abscess and hydromyelia due to a late diagnosis, thereby worsening the spinal cord injury.

“Syrinx” and “syringomyelia” are synonymous and are defined as a fluid-filled cavity located inside the spinal cord. When this cavity is located in the central canal it is called hydromyelia [6]. Syringomyelia occurs in approximately 25% of occult spinal dysraphism cases: meningocele manqué, diastematomyelia, and rarely other anomalies as in the described case of DST [7]. In 70% of syringomyelia, cases are related to abnormal CSF circulation in the cranial or spinal subarachnoid space [6]. In the case reported, there was a CSF flow dynamics disturbance due to the infectious process.

The clinical picture is usually non-specific, with fever, meningism, neurological deficit, and bladder incontinence. There is also secretion flow through the ostium of the congenital dermal sinus associated with the infection. When a spinal cord abscess occurs, there are some differential diagnoses, such as transverse myelitis, Guillain-Barré syndrome, and neoplasms. Spinal cord abscess is the most serious complication, and the most frequent causal organism is *S. aureus* [2,5].

MRI is the gold standard in neuroimaging investigations because it allows for the identification of DST and the extent of infection, whether epidural, subdural, or intramedullary [5]. Guillain-Barré syndrome and neoplasm were initially considered differential diagnoses in this case due to the non-specific clinical picture. However, the diagnosis of a dermal sinus became more evident after MRI.

Congenital dermal sinus treatment has a better prognosis in cases diagnosed as early as possible, allowing surgery to be performed while the tissue is not infected and lesions are minor, thereby preventing future infections. In cases of late diagnosis with abscess formation, surgical drainage and appropriate antibiotic therapy are recommended to avoid irreversible damage to the spinal cord due to vascular involvement and the effects of mechanical pressure. Unfortunately, delays in diagnosis and intervention complicate the surgical procedure, with a significant risk of neurological deficits. Thus, surgery involves the complete removal of DST, drainage of the abscess with the possible removal of its wall, resection of any associated inclusion tumors, and obtaining samples for microbiological and histopathological examinations [8]. In the present case, in addition to the resection of DST, it was also possible to obtain these samples for examination. The abscess was drained, and syringomyelia was also treated, restoring the CSF flow. Sequelae persisted even after the surgery because of the late diagnosis, and the child already had a severe neurological deficit.

## Conclusions

This report described a case of DST that showed a rare association of two complications, i.e. abscess and hydromyelia, due to late diagnosis. Despite the surgical procedure, sequelae still persisted.

As in the reported case, DST can evolve with complications and early diagnosis starting with a good physical examination can avoid sequelae.
